# Classification of Tennis Shots with a Neural Network Approach

**DOI:** 10.3390/s21175703

**Published:** 2021-08-24

**Authors:** Andreas Ganser, Bernhard Hollaus, Sebastian Stabinger

**Affiliations:** 1 Department of Mechatronics, MCI, Maximilianstraße 2, 6020 Innsbruck, Austria; andreas.ganser@mailbox.org; 2 Deep Opinion, 6020 Innsbruck, Austria; sebastian@stabinger.name

**Keywords:** deep learning, wearable computing, activity recognition, tennis shot classification

## Abstract

Data analysis plays an increasingly valuable role in sports. The better the data that is analysed, the more concise training methods that can be chosen. Several solutions already exist for this purpose in the tennis industry; however, none of them combine data generation with a wristband and classification with a deep convolutional neural network (CNN). In this article, we demonstrate the development of a reliable shot detection trigger and a deep neural network that classifies tennis shots into three and five shot types. We generate a dataset for the training of neural networks with the help of a sensor wristband, which recorded 11 signals, including an inertial measurement unit (IMU). The final dataset included 5682 labelled shots of 16 players of age 13–70 years, predominantly at an amateur level. Two state-of-the-art architectures for time series classification (TSC) are compared, namely a fully convolutional network (FCN) and a residual network (ResNet). Recent advances in the field of machine learning, like the Mish activation function and the Ranger optimizer, are utilized. Training with the rather inhomogeneous dataset led to an F_1_ score of 96% in classification of the main shots and 94% for the expansion. Consequently, the study yielded a solid base for more complex tennis analysis tools, such as the indication of success rates per shot type.

## 1. Introduction

In society, interest is growing in monitoring physical performance in everyday life as well as in sports. Sales of wearable devices, such as fitness trackers or chest straps, have been growing tremendously over the last decade [1]. The mainstream solutions focus on supervising heart rate and motion recognition (e.g., step counters or position tracking with the help of inertial measurement units (IMU) and global positioning systems (GPS) [2]). As stated in [3,4,5,6,7], IMUs, in particular, are frequently used to collect information about training progress and general sports analytics. Analysing this data helps with improving the training specificity and preventing injuries [8,9].

For training purposes on a competitive level, more advanced sport-specific solutions are needed. In swing based sports, such as tennis, badminton, and squash, the shot performance is valuable information to develop better training and game plans. How interesting would it be if the worn smartwatch could tell the tennis player how fast their fastest service was during the last match? If this information is combined with the success rate of the respective shot type, insights for the next training session could be obtained. The prerequisite for such a sophisticated analysis is the reliable detection and classification of tennis shots, which is the topic of this study.

### 1.1. Market Analysis

The market already provides several solutions for tennis shot analysis. They can be grouped into three categories:

(1) Camera-based analysis tools, such as PlaySight [10], have a high shot recognition rate and can enable detailed evaluations depending on the complexity of the algorithm. The drawback of this technology is its high price [11]. Hence, these systems are not widespread and are mostly used by players who are on a professional level. Vision recognition tools are not further considered in this study since the solution should, in the long run, be available for a broad audience.
(2) Racket integrated solutions, provided by tennis racket manufacturers, are cheaper than the previous technology, but lack in recognition accuracy [11]. An associated study [12] using the Pan Tompkins algorithm for shot detection and time warping for shot classification achieved an accuracy close to 96%. Nevertheless, the sensors were fixed to a racket and were, therefore, non-mobile. Additionally, the recognition of topspin and backspin has an accuracy of only 80%. Furthermore, the attachment of sensors to the racket changes the fine-tuned centre of mass.
(3) Wrist-worn wearables, using the dynamic time warping (DTW) algorithm [13,14], can achieve a shot classification accuracy of up to 99%, but remain close to 80% for topspin and backspin detection [15]. Another technology for wrist wearables compared neural network approaches to feature recognition and reached a success rate of 94% for groundstrokes [16]. A study published in 2017 by [17] generated data with an IMU, worn at the wrist, and also compared several approaches for shot classification. In general, the rather classical support-vector machine (SVM) performed best with an accuracy of 97.4% for the groundstrokes, specifically the forehand, backhand, service and false shot. Whiteside et al. also implemented a nine-shot type classifier with a mean accuracy of 93.2%. The SVM classifier distinguishes between forehand topspin, slice and volley; backhand topspin, slice and volley; serve; smash; and false shot. The support vector machine is followed by a deep neural network classifier, reaching an accuracy of 96.6% for the four groundstrokes. The extended version reaches 90.4% for the nine shot types classifier.

State-of-the-art deep neural networks are well suited for time series classification (TSC) [18] and give new possibilities in classifying tennis shots. Unfortunately, [17] does not give deeper insight into the creation and application of the classifier. As the literature analysis revealed, there are currently few tennis shot recognition solutions with a deep neural network classifier at the core since this combination is relatively new.

### 1.2. Biomechanics in Tennis

For a better understanding of the sensor signals, shown in Section 2.1, it is vital to understand tennis shots anatomically. The focus lies on the upper limb—more specifically the shot hand. The movement of the upper extremity in tennis sports can be described as a combination of four basic motions [19]:

1. Pure swing of the upper arm around the shoulder joint: ground swing.
2. Elbow joint flexion and extension: increases the swing.
3. Forearm pronation and supination: rotation around the forearm longitudinal axis, responsible for the topspin or backspin.
4. Wrist extension and flexion: tilt of the wrist, also increases the swing.

**Figure 1:**
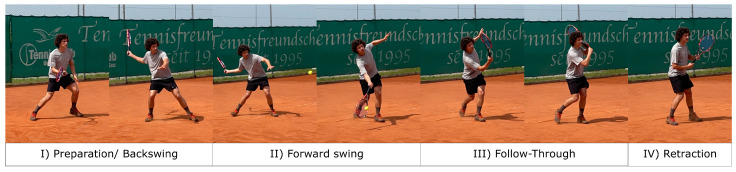
Sequence of a tennis forehand, subdivided in four parts.

Additionally, Ref. [19] separates tennis shots into several sequential stages, which are outlined on the example of a forehand shot in Figure 1:

(I) Preparation/Backswing: the hand starts at resting position, throws the ball up; at the same time, the racket is guided upwards and down behind the back with a flexion of the shoulder and the elbow joint; the phase finishes when the racket reaches the lowest point.
(II) Action phase/Forward swing: the shot forearm and shoulder joint are extended; the racket is guided upwards and forwards; the impact of ball and racket ideally occurs at the highest point, so fully extended elbow and wrist, arm showing upwards.
(III) Follow-through: after the impact, the kinetic energy of the movement has to be dissipated, which is done by letting the momentum run out by swinging the shoulder through; usually, the racket stops at a very low point.
(IV) Retraction: bringing the shot hand back into a neutral position to be ready for the next shot.

These four phases are present in all tennis shot types, but differ in the combination of the anatomical motions, which results in distinguishable sensor signals. The tennis shots are categorized into three groundstrokes, which are expanded with the spin to five shot types in total and are described in Table 1.

Slice and volley are combined into one shot as the motion is very similar. The same applies to service and smash, which are anatomically the same movement with a different location on the court.

## 2. Methods

### 2.1. Shot Detection

To enable shot detection in tennis, a platform to gather data of the shots is needed and should provide data containing information on the shot type. Other sports have used wearables successfully to gather such data [20,21,22]. We also used this approach using wearables in this paper.

#### 2.1.1. Hardware

**Figure 2:**
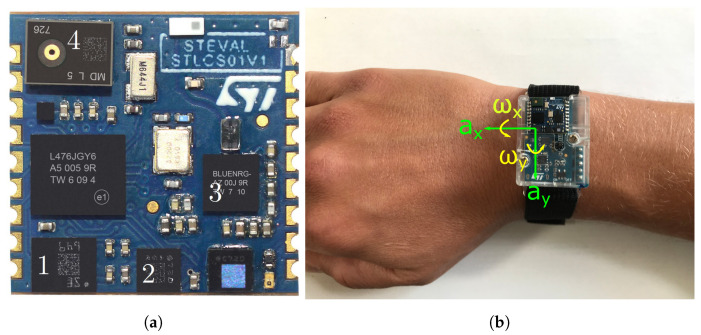
Sensor tile displayed as (**a**) the board itself with numbered sensors according to Table 2, adapted from [24], and (**b**) the complete wearable with marked sensor axes, worn on the wrist. The axes for the accelerometer $a_{x}$, $a_{y}$, and the gyroscope $\omega_{x}$, $\omega_{y}$ are displayed.

The wearable used for recording the dataset was the SensorTile development kit (STEVAL-STLKT01V1) of STMicroelectronics, Geneva, Switzerland, which is illustrated in Figure 2 and includes the sensors mentioned in Table 2. The development kit is chosen for the tennis shot detection task since it has already proved its abilities in a catch detection application for American Football [20]. Additionally, the sensor kit comprises all relevant sensors to monitor motion, pressure, and audio in satisfying sample rates and ranges, which is key for a later classification.

**Table 2:** Sensor properties as set on the development kit. Recording sensor, output data rate (ODR) and full scale (FS) are assigned to the respective signal. For more information, we refer the reader to the relevant datasheets [23,24,25,26,27,28,29].

No.	Signal	Sensor	ODR	FS
1	Acceleration *a*	LSM6DSM	1660 Hz	156.96m/s^2^
1	Angular velocity ω	LSM6DSM	1660 Hz	2000°/s
2	Magnetic field *B*	LSM303AGR	100 Hz	49.152 G
3	Pressure *p*	LPS22HB	75 Hz	1260 hPa
4	Quantized audio signal	MP34DT05-A	8000 Hz	122.5 dBSPL

#### 2.1.2. Shot Detection Algorithm

**Figure 3:**
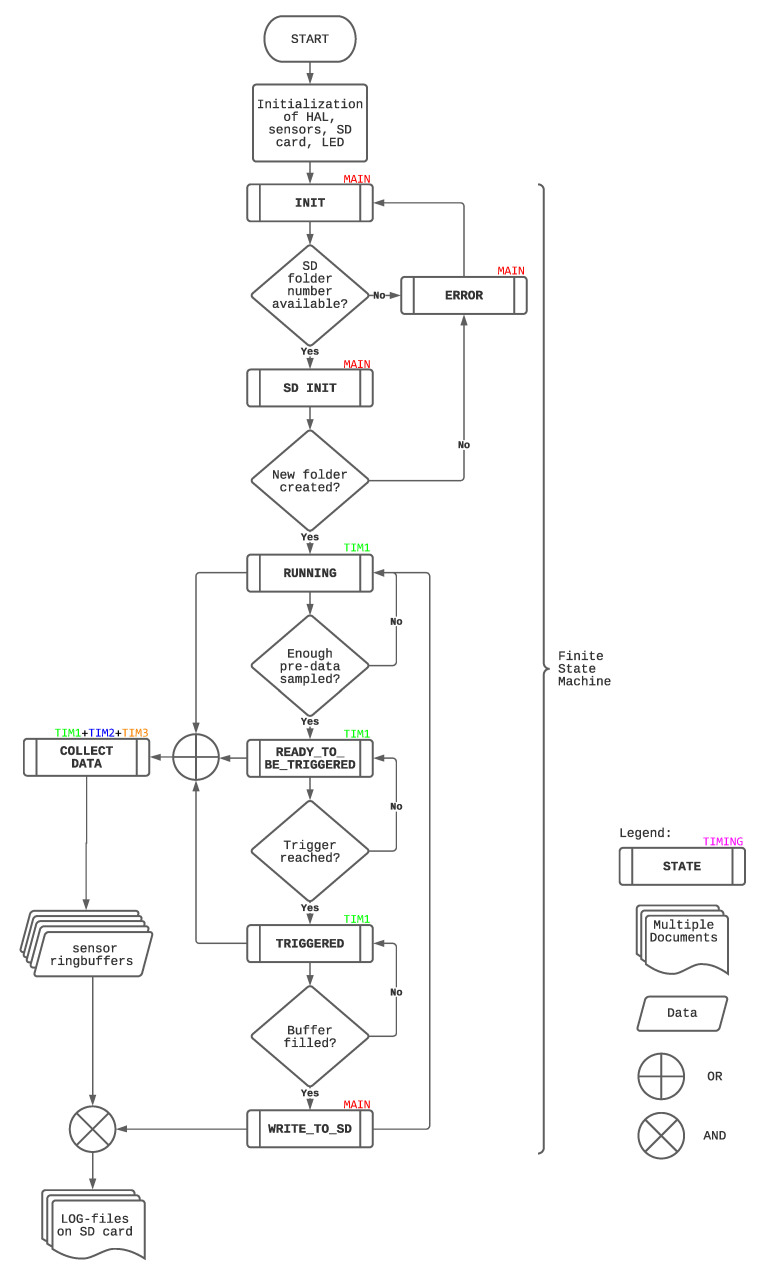
Flowchart of the state machine running on the microcontroller. Note that there is an annotation about the location of the state.

The shot detection algorithm is implemented in the programming language *C* with a finite state machine (FSM) [30]. The FSM, designed for shot detection, is visualized in Figure 3 and is composed of eight states. These states are implemented in the main as well as three timers. Figure 3 shows not only the sequential process but also where the state is realized.

For example, the triggering procedure, responsible for recognizing the tennis shots, is located in timer 1 (TIM1), which runs with 1 kHz. Triggering is done in the states RUNNING, READY_TO_BE_TRIGGERED, and TRIGGERED and is further described in Section 2.1.3. The basis for triggering is the accelerometer and gyroscope data, which are saved as signals in circular buffers [31]. The magnetometer and the pressure signal are collected in TIM2, which runs with 100 Hz, since the ODRs of the respective sensors do not allow faster sampling. An exception is the audio data which is gathered in TIM3 with the highest sampling rate, namely 8 kHz, to capture all the expected frequencies during a tennis shot.

**Figure 4:**
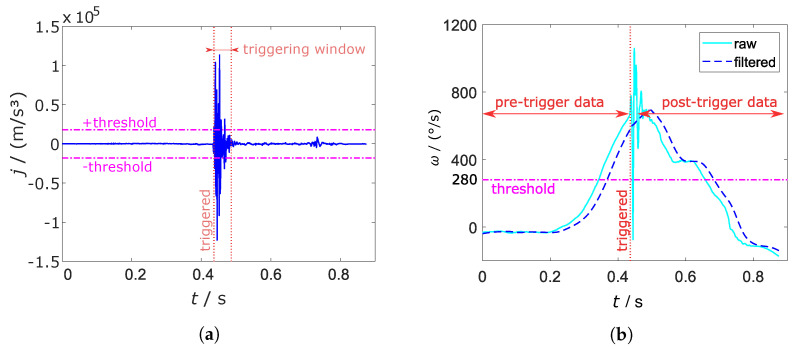
Jerk *j* (**a**) and angular velocity in y-direction $\omega_{y}$ raw and filtered (**b**) of a forehand topspin compared to the trigger thresholds. The time delay of the filtered $\omega_{y}$ is clearly visible.

Responsible for accessing the sensor signals and writing them into the respective circular buffer is the state COLLECT_DATA, which, therefore, has to run in all the above mentioned timers. This state is active during the states RUNNING, READY_TO_BE_TRIGGERED and TRIGGERED since samples have to be collected before and after triggering to save the complete shot sequence as mentioned in Section 1.2. Several sensor data plots, like Figure 4a,b show that 1 s is sufficient to cover the whole shot. Furthermore, the plots reveal that the buffer has to be filled with 500 ms of data before and after the trigger.

#### 2.1.3. Triggering

Searching for an adequate trigger, which is responsible for recognizing tennis shots and, therefore, starting the saving process of the sensor values, is one key aspect of this study. A selective trigger decreases the post-processing effort, since less falsely detected shots have to be discarded. Optimally, it captures every performed shot, corresponding to a high sensitivity. Since there is a conflict between sensitivity and selectivity [32,33], a suitable trigger algorithm has to be found.

The trigger is realized with two components. A combination of a value modelling the impact of the ball on the racket and another value representing the specific swing performed during tennis shots is chosen. In this way, the balance between falsely detected shots and undetected shots is optimized. On the one hand, the final trigger must capture all types of shots named in Section 1.2. On the other hand, several scenarios are considered that should not be detected:

1. A player hitting his racket on the ground to pick up a ball: high impact, low swing.
2. A player swinging his racket without hitting a ball: low impact, high swing.
3. A player sprinting or jumping: mid impact, low swing.

All in all, three triggering solutions are investigated; however, only the finally implemented method is described in more detail. The other two approaches are accessible in Appendix A.1 and Appendix A.2.

The jerk is chosen as the adequate parameter for the impact of the ball on the racket. The jerk is the change rate of the acceleration with respect to the time. The acceleration is changing with a high frequency when the ball hits the racket, consequently, with a high rate of change. Figure 4a shows the high lobes of the jerk during a forehand topspin. The derivative is taken from the absolute acceleration because the combined signal shows higher peaks during the vibrations of the racket.

We empirically determined that a jerk threshold of 18,000 m/s^3^ led to reliable triggering. The threshold is compared to a forehand topspin in Figure 4a.

**Figure 5:**
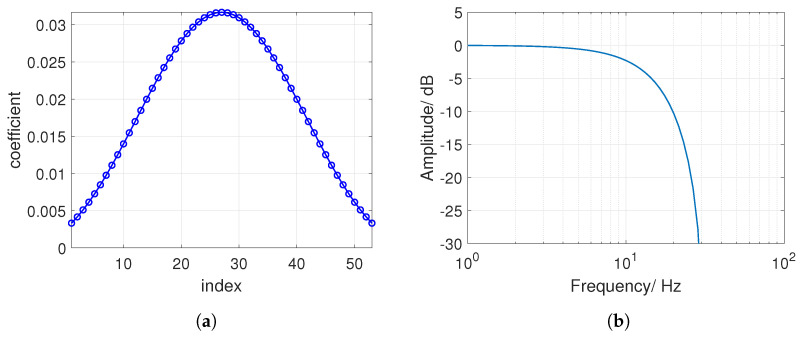
(**a**) FIR filter coefficients and (**b**) frequency response of the FIR filter.

The angular velocity around the *y*-axis $\omega_{y}$ might be a suitable representative of the pure swing components as is illustrated in Figure 4b. It exhibits a high peak for all shot types. Nevertheless, $\omega_{y}$ is also high during shocks which arise, for example, when running or hitting the racket on the ground. Hence, the threshold is compared to a low-passed $\omega_{y}$ signal. The finite impulse response (FIR) filter is designed to cut frequencies higher than 15 Hz with an order of N=53. It is designed with a Kaiser window function. The filter coefficients and the magnitude response is illustrated in Figure 5.

As a consequence, the vibrations caused by hard hits or the impact of the ball are vanished as can be seen in Figure 4b. This configuration adds an delay $t_{d}$ to the sensor signal according to

(1) $$t_{d}=\frac{N-1}{2f_{s}},$$

with $f_{s}$ as the sampling frequency. Calculation with N=53 and $f_{s}=$ 1000 Hz yields a delay $t_{d}=$ 26 ms, which is still in an acceptable range. The threshold is set to 280 ${}^{\circ }$s^−1^ and can be seen in Figure 4b.

Due to the delay of the filter, a window with a size of 50 ms is implemented. Both thresholds must be exceeded in the latter; otherwise, the trigger is not set. Figure 4a shows the triggering window for the first overshooting of the threshold. The window is restarted after the threshold is surpassed again.

### 2.2. Generation of the Dataset

Data was collected during training and games of players on a mainly competitive amateur level. In total 16 players, 6 male and 10 female, from an age of 13 to 70 years old wore the wristband to cover a wide range of playing styles. The participants were informed about the MCI ethics assessment and signed a declaration of consent. Additional to the data collection with the wristband, a camera was used to record the session and to label the datasets later on.

**Figure 6:**
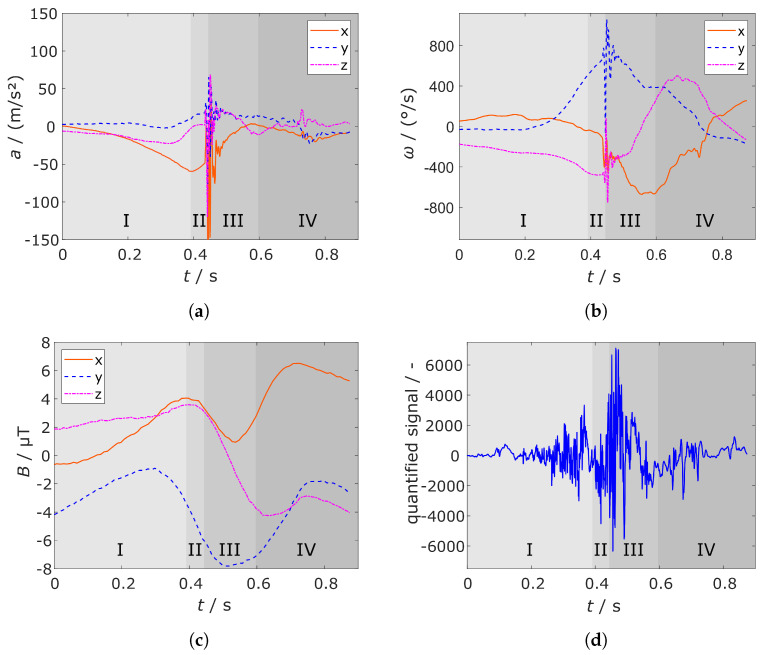
Visualisation of the 10 sensor signals of a forehand topspin with annotated sequences as mentioned in Section 1.2: (**a**) *x*, *y*, *z* component of the accelerometer, (**b**) *x*, *y*, *z* component of the gyroscope, (**c**) *x*, *y*, *z* component of the magnetometer, and (**d**) quantified audio signal.

Before being able to use the data for training and validating the shot classifier, some pre-processing was performed on the datasets. Neural networks require a feature vector or, in this case, a tensor as input with all entries having the same amount of samples, but the collected sensor buffers have different lengths because of the varying sampling frequencies mentioned in Section 2.1.1. Therefore, the missing sensor samples were interpolated linearly to match the amount of samples of the audio signal. Moreover, the pressure signal did not show a remarkable change whenever a shot was performed. This, and the fact that it was only sampled with a frequency of 100 Hz led to the decision to exclude the pressure data from the dataset. The remaining ten sensor buffers, which are displayed in Figure 6, were extended with the shot hand information encoded as dummy values.

The resulting input feature tensor has a dimensionality of 11×7000 and consists of Z-Score normalized values. The Z-Score of each sample is derived according to [34]:

(2) $$z_{i}=\frac{x_{i}-\mu }{\sigma },$$

with *x* as the current sensor value, σ as the standard deviation, and μ as the arithmetic mean value of the respective shot and sensor.

The output feature tensor contains the one-shot encoded shot type information. After the labelling process, the datasets are anonymised by shuffling them several times and renaming them incrementally.

### 2.3. Shot Classification with a Deep Convolutional Neural Network

Deep neural networks (DNN) have shown especially promising results in speech recognition [35] and natural language processing (NLP) [36]. NLP and speech recognition have the sequential aspect of the data in common, which is also an important feature of the time series data processed in this study. The authors in [37] saw this as an opportunity to research deep neural network performance regarding TSC problems. One main question of his review was whether DNNs could surpass standard classification processes, like the hierarchical vote collective of transformation-based ensemble (HIVE-COTE) [38] or dynamic time warping (DTW) [13,14] as used in a tennis shots classification approach by [15], in terms of computational effort and classification accuracy.

Based on the research in [37], the two best-performing architectures were adapted for the classification problem of tennis shots. The best performers, namely a fully convolutional network (FCN) and a residual network (ResNet), are categorized as discriminative end-to-end approaches [39,40,41]. End-to-end models do not require any hand-engineered features of the input training data. The particular architectures learn the feature extraction on their own while fine-tuning the classifier in the backpropagation process [42,43].

#### 2.3.1. Architecture of the FCN

**Figure 7:**
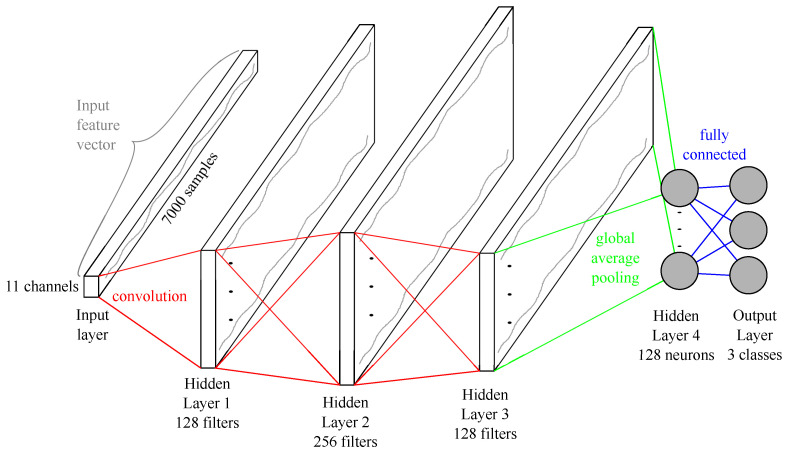
Schematic visualization of the FCN architecture for the three classes model.

FCNs were first presented for a time series classification problem in 2016 by [44]. The FCN for the shot classification is built with four hidden layers, and the input and output layer. The main components are the three convolution blocks. The first convolution consists of 128 filters with a length of eight; the second contains 256 filters with a filter length equal to five. The last convolution reduces the number of filters back to 128 and the filter length to three. Every convolution is pursued by a batch normalization [45]. The output of the batch normalization is fed into a Mish activation function [46]. After the third convolutional block, a global average pooling (GAP) layer [47] is applied, followed by a softmax operation [48]. Furthermore, the length of the time series is kept constant with adequate zero-padding until the GAP layer. Figure 7 shows the complete architecture of the FCN.

#### 2.3.2. Architecture of a ResNet

Residual networks, first published in an image classification competition in 2015 by [49], are convolutional networks with up to 1000 layers that are still trainable. This deepness is made possible by the so-called “identity shortcut connections”, which skip one or more layers [50]. Via these connections, the gradient can flow backwards unimpeded. Thus, the vanishing gradient problem is reduced, making it possible to use deeper networks that can mimic more complex functions.

In 2016, the researchers in [44] released a relatively deep ResNet for time series classification. This architecture consists of the indispensable input layer, nine convolutional layers, and one GAP layer that is fully connected to the output layer with the classical softmax activation. The nine convolutional layers are dividable into 3×3 blocks of similar structure: The first of these three blocks consists of three convolutions with 64 filters of size eight, five, and three. Each convolution is followed by batch normalization and the Mish activation function, apart from the last one.

**Figure 8:**
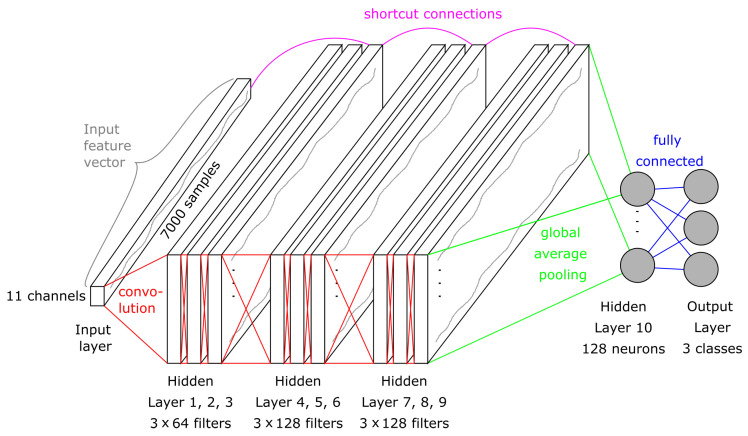
Visualization of the ResNet architecture for the three shot types classification. The first 9 hidden layers are divided into 3 × 3 similar blocks. Each block has a shortcut connection to the previous one.

After the third filter and batch normalization, the interim result is added to the identity of a shortcut connection. The sum is activated with a Mish function and then fed into the next block. The consecutive blocks differ only slightly. The amount of filters is increased to 128, the rest is kept as before. The shortcut connections take the output of the latter block instead of the input layer. For a better understanding, the architecture is visualized in Figure 8.

#### 2.3.3. Training of the Deep Neural Network Classifiers

The creation of the classifiers is implemented in Google Colaboratory [51], which is a cloud service based on Jupyter Notebooks [52]. It offers a free-of-charge use of a graphics processing unit (GPU) such as an NVIDIA Tesla T4, (NVIDIA, Santa Clara, CA, USA), which outperforms standard central processing units (CPU) by far [53]. Training sessions of the tennis shot classification are executed around 25–30-times faster. Another reason for the use of Google Colab is the out-of-the-box support of the open-source deep-learning library Keras [54], which runs on Tensorflow [55] as a backend.

**Figure 9:**
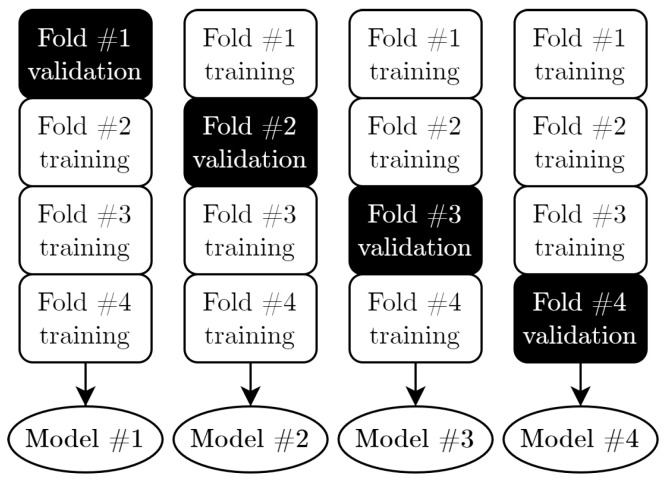
Operating principle of the K-Folds cross validator. The shuffling of the stratified sets leads to dissimilar models per iteration.

A successful training is strongly dependent on the quality of the training and validation set. An important measure is that all the classes are represented as equally as possible in all sets. The used stratified K-Folds cross validator [56] splits the dataset into n folds and preserves the percentage of samples for every class. For this application, four folds are created, meaning that four different models are trained. Figure 9 illustrates the operating principle of stratified K-Folds, which swaps the training and validation sets for every iteration. The fact that more than one model is created allows creating averages and standard deviations of several metrics for checking the real capability of the model, independent from the weight initialization.

As an optimizer, Ranger [57] is used. Ranger is a combination of three algorithms, namely RectifiedAdam (RAdam) [58], Lookahead [59] and Gradient Centralization (GC) [60]. Ranger is not yet implemented in TensorFlow nor in Keras. Nevertheless, the documentation of RAdam proposes the integration of the Lookahead optimizer to generate the Ranger optimizer. This modification is used for the shot classification training. The GC add-on is left for future work.

**Figure 10:**
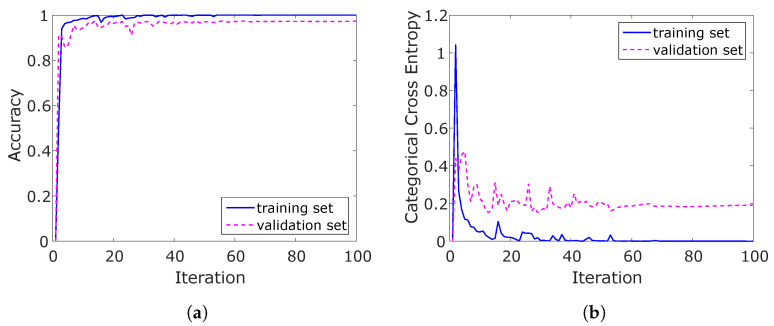
Training history of (**a**) accuracy and (**b**) categorical cross entropy (CCE).

Another critical question is the training time, more specifically, how many training epochs should be used. An exemplary training session is displayed in Figure 10. The training finished here after 130 epochs, and the results were stable after 60. The training epochs are fixed to this empirically determined value. The duration of this exemplary training process was 13 min, resulting in 6.1 s per epoch. In conclusion, the settings mentioned above resulted in stable behaviour.

## 3. Results

### 3.1. Shot Detection Trigger

The setup described in Section 2.1.3 yielded a 91% success rate of shot detection. The other investigated solutions were abandoned due to the reasons mentioned in Appendix A.3.

False positives were very rare, only 2%. The trigger was not set in situations that are closely related to a shot, for example, when a player picks up the ball from the ground by hitting it several times. However, the time intensive data saving, which takes nearly 2 s, is the reason why quick consecutive shots were not captured, for example when one player was at the net and playing volleys. Furthermore, in rare cases, the backhand slice was not detected because of the unfavourable orientation of the wearable that resulted in a lower $\omega_{y}$ value.

### 3.2. Dataset

**Figure 11:**
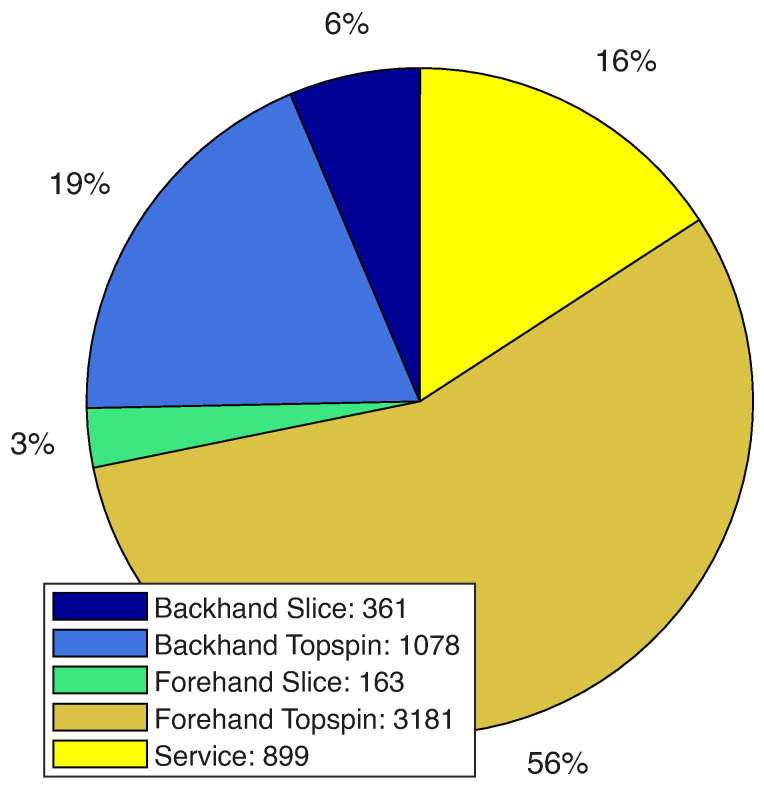
Distribution of the shot types in the final training set.

Overall video material of 18 h resulted in 5682 labelled tennis shots. The distribution over the shot types is illustrated in Figure 11. The slice version of the shots is, with 6.35% for backhand and 2.87% for forehand, significantly under-represented, although volley and slice are already combined. For the groundstroke dataset, the two backhand types were combined, resulting in 1439 shots that had a 25% contribution to the overall training set. Additionally, the forehand shots were merged, which yielded 3344 shots or 59%. Services were the same as before. Another statistic is the division of the dataset into left and right-handed players. Here, left-handed shots are represented with only 7%.

### 3.3. Shot Classification

The final results of the classifiers are shown for the three classes networks and five classes networks. First, the three classes model is compared for the FCN and ResNet. Second, the result of the five classes network is only shown for the ResNet, because of the reasons mentioned in Section 3.3.1. All following metrics are introduced in [61].

#### 3.3.1. Three Shot Types Classification

**Figure 12:**
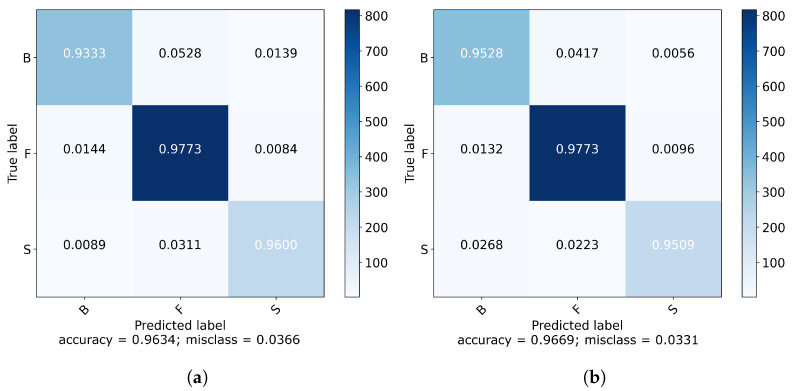
Normalized confusion matrix for the FCN (**a**) and the ResNet (**b**) comparing the three classes: backhand (B), forehand (F), and service (S).

The normalized confusion matrices of the respectively best iterations in Figure 12 indicate strong diagonals.

Table 3 shows the results in more detail. The F_1_ score for all shot types is in the range of 94–97%. The recall and precision are constantly high with values between between 93–98%.

Both architectures are well suited for the classification task with the ResNet having a slightly better performance. Additionally, the optimization of the ResNet architecture reaches a stable state on average five epochs faster. Moreover, forehand seems to be a little easier to predict. The reason might be the higher representation of forehands in the dataset.

#### 3.3.2. Five Shot Types Classification

The results of the five classes models are presented only by the ResNet. There is merely a slight difference in accuracy to the FCN, but the ResNet trained faster.

**Figure 13:**
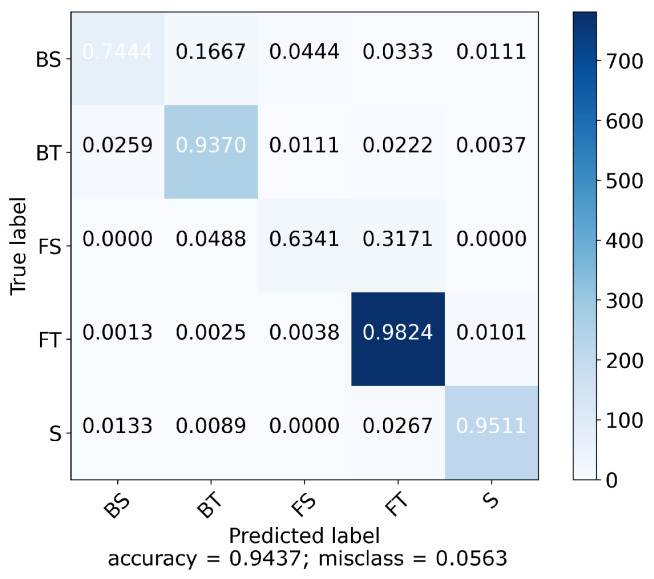
Normalized confusion matrix for the best ResNet comparing the five classes: backhand slice (BS), backhand topspin (BT), forehand slice (FS), forehand topspin (FT), and service (S).

The confusion matrix in Figure 13 has high percentage values in the diagonal for the topspin versions and the service, i.e., the standard shots. It has to be mentioned that the slice variants are less accurately categorized. The wrongly classified samples tend to be misclassified into the topspin equivalent or as the other ground shot’s topspin. Hence, the model is overfitted to the ground shots.

Table 4 illustrates the main metrics for an inhomogenous dataset problem. The results of the confusion matrix are confirmed. The average is very high, with a percentage of 94%. The reason for this is the dominating influence of the forehand topspin with nearly 56% contribution to the whole dataset. Hence, also the low recognition rate of the forehand slice effects the sample average of the F_1_ score only a little, as it represents only 2.9% of all samples.

## 4. Discussion

### 4.1. Shot Detection Trigger

The shot detection trigger is accurate for the groundstrokes. Improvement lies in the recognition of the slice variants due to the unfavourable orientation of the wearable. A possible solution could be a more complex algorithm which includes another trigger value as representative of slice shots. The data saving time is another bottleneck which does not allow to capture quick consecutive shots. With these two enhancements, a detection rate of around 95% can be expected, which lies in the range of the best published research thus far [12]. As the focus of this study is more shifted towards the generation of a classifier, the reached accuracy is considered to be sufficient, and optimizations are left for future work.

### 4.2. Shot Classification

Compared to other approaches, such as dynamic time warping and support vector machines, mentioned in Section 1, this study reached higher classification accuracies for the groundstrokes apart from the one mentioned in [15]; however, the less complex and more module solution justifies the worse recognition rate. The additional distinction between slice and topspin worsened the performance emphasizing the importance of a homogeneous dataset.

Furthermore, the classification success was decreased by the limitations of the sensors. The full scale values of the gyroscope ( 2000 ${}^{\circ }$s^−1^) and the accelerometer ( 156.96 ms^−2^) were exceeded with fast shots. The clipping at the borders adds non-linearities to the sensor signals. Sensors with a wider measurement range would allow the gathering of more precise data and could consequently improve the classification accuracy.

#### 4.2.1. Validation of the Dataset Quality

**Figure 14:**
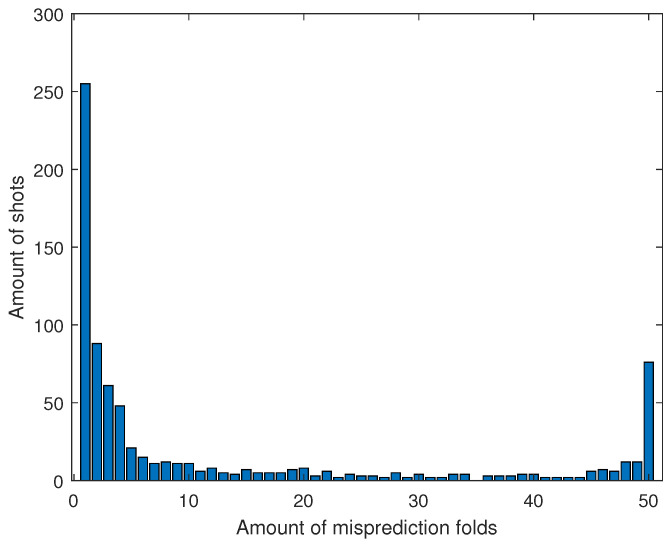
The *x*-axis gives the amount of folds in which a shot is mispredicted. The *y*-axis is the amount of shots that occur in the respective bin.

Mislabelled datasets can be a reason for falsely classified shots. During a training session with 50 iterations, the mispredicted shots were tracked and their unique identifiers noted. A stratified K-Fold in each iteration ensured that every shot is 50 times in the validation set. Figure 14 shows how often a shot is falsely classified in how many iterations. If a shot is incorrectly classified in all fifty iterations, the probability is high that the label is wrong or it is a complex shot and, therefore, hard to classify.

Note that the histogram in Figure 14 is having its peak at the first two bins. This indicates that the network is not able to predict these shots in individual iterations. The uncertainty in the weight distribution of the network is held responsible for this. However, in 76 of the 5682 shots, located on the far right of Figure 14, the current setup is not capable of training the DNN to predict certain shots correctly. These are 1.34% of the total dataset. These shots are either labelled incorrect or seldom occurring and, therefore, hard to train.

#### 4.2.2. Ablation Study

An ablation study [62] is performed for the three-class ResNet to receive information about the significance of the input values. The objective is to optimize the input feature tensor with a simultaneous improvement of the neural network and a possible downsizing of the sensor board. The mean F_1_ score over four iterations for every configuration is compared. The sensor values, which are highlighted with a checkmark in Table 5, are left unchanged, whereas the others are filled with zeroes instead of real values.

Interestingly, the ablation study indicates an independence of the DNN classifier from the audio data. In general, the audio data can be excluded from the feature tensor and, consequently, also the microphone on the sensor board. Note that the audio data is sampled with 8 kHz to capture all the necessary information. If the audio data is not included, the feature tensor can be reduced considerably. Consequences are a smaller dataset and network yielding a faster training of the latter.

## 5. Conclusions and Future Work

This study found that a deep neural network approach reached high accuracies in tennis shot classification when a rich, homogenous dataset was used. The generation of the latter one is difficult to obtain when only taking data from games or training sessions since the groundstrokes are always overrepresented. Data augmentation, including averaging, amplification, dynamic time warping, addition of noise, etc. [37,63,64] is a possibility to smooth the distribution over the shot types, but was not considered in this study.

Nevertheless, high classification rates were achieved with a rather inhomogenous data set. Recent developments in the architecture of deep learning networks and the newest research on more stable activation functions and optimizers made this possible.

Furthermore, the results show another capability of deep convolutional neural networks for time series classification. The generation of a dataset can be done with much less domain knowledge because no striking features have to be extracted. Therefore, the pre-processing effort was reduced drastically.

Triggering with a combination of filtered angular velocity $\omega_{y}$ and jerk *j* yielded a reliable detection rate. Since the focus of this study was shifted toward the classification process, this result is considered sufficiently accurate. The reliability of the triggering decreased the post-processing effort for labelling the shots, as only few false positives were detected.

One recommended next step should involve the development of a wearable whose sensors have adequate full scales. Consequently, the classification accuracy could improve since the sensor signals will not be clipped. Future work can also focus on better analysis functions. One suggestion is the development of a real-time shot classification to directly see information about playing styles—for example, during training sessions or games. The information could be available in a smartphone application. The used wearable already has a BlueTooth module, which could be used for the transmission of the data. Valuable information for the players would also be the quality of the shot and the success rate per shot. For this purpose, another dataset must be generated. The position of the ball on the surface of the racket during impact and the success of the shot must be labelled for this.

Furthermore, an implementation of the wearable into a smart-watch would be a step to create a product that could be offered to a broader audience.

## Figures and Tables

**Table 1:** Division of the tennis shots separated into groundstrokes and their expansion. The abbreviation for the respective shots is also noted.

Groundstrokes	Expansion
Forehand (F)	Topspin (FT)
	Slice (FS)
Backhand (B)	Topspin (BT)
	Slice (BS)
Service (S)	Service (S)

**Table 3:** Recall (R), precision (P), and F_1_ score (F_1_) for FCN and ResNet for the respective shot types of the best model.

	FCN			ResNet		
	R	P	F_1_	R	P	F_1_
B	93%	96%	95%	96%	94%	95%
F	98%	96%	97%	98%	97%	98%
S	93%	94%	94%	93%	96%	95%
Average	96%	96%	96 %	96%	96%	96%

**Table 4:** Recall, precision, and F_1_ score for ResNet for the respective shot types of the best model.

	R	P	F_1_
BS	77%	82%	80%
BT	92%	94%	93%
FS	76%	68%	72%
FT	97%	97%	97%
S	94%	90%	92%
Average	94%	94%	94%

**Table 5:** Ablation study for the input values of the three-class ResNet.

Accelerometer	Gyroscope	Magnetometer	Audio	F${}_{1}$-Score
✓				95.8%
	✓			95.3%
		✓		95.3%
			✓	69.3%
✓	✓	✓		96.6%
✓	✓		✓	96.0%
✓		✓	✓	96.1%
	✓	✓	✓	95.8%
✓	✓	✓	✓	96.4%

## Data Availability

Samples and code are available from the authors.

## Abbreviations

The following abbreviations are used in this manuscript:

CNN	convolutional neural network
CPU	central processing unit
DNN	deep neural network
DTW	dynamic time warping
FCN	fully convolutional network
FIR	finite impulse response
FS	full scale
FSM	finite state machine
GAP	global average pooling
GPS	global positioning system
GPU	graphics processing unit
HIVE-COTE	hierarchical vote collective of transformation-based ensemble
IMU	inertial measurement unit
MCI	Management Center Innsbruck
NLP	natural language processing
ODR	output data rate
ResNet	residual network
SVM	support vector machine
TSC	time series classification

## Appendix A. Alternative Triggering Methods

Additional to the triggering method described in Section 2.1.3, two more solutions were investigated on a small scale. Nevertheless, the authors want to clarify the decision to use the finally implemented trigger by explaining the other approaches as well.

### Appendix A.1. Audio

The idea of triggering with the microphone data was to capture the distinct sound of the moment when the ball hits the racket. Nevertheless, the audio signal as a trigger failed because of the disturbing wind generated by the fast movements. The noisy audio signal of a forehand topspin is shown in Figure 6d. The exact moment of the racket hitting the ball is not unambiguously recognizable. Trials to protect the device from the wind with a pop filter [65] were not successful, and therefore, this solution was abandoned.

### Appendix A.2. Variation of the Filtered Net Rotational Energy and Jerk

Since tennis is a swing sport, the rotational energy during a shot is relatively high, resulting in a significant lobe in the $\omega_{energy}$. Hence, the second idea was the triggering via a modification of the net rotational energy $E_{\omega }$, which is defined as

(A1) $$E_{\omega }=\frac{I\omega^{2}}{2}$$

where *I* is the moment of inertia of a body around its rotational axis [16]. Therefore, also

(A2) $$E_{\omega }\propto \omega_{energy}^{2}=\omega_{x}^{2}+\omega_{y}^{2}+\omega_{z}^{2}$$

holds true. This result is smoothed with the filter specified in Section 2.1.3 and compared to an empirically determined threshold value.

This swing representative is combined with a threshold for the jerk in the same way as mentioned in Section 2.1.3.

### Appendix A.3. Comparison of the Triggers

Table A1 gives an overview about the trigger possibilities. In the end, the $\omega_{y}+j$ combination was chosen due to its higher selectivity in comparison to the $\omega_{\mathrm{energy}}+j$ solution.

**Table A1:** Advantages and drawbacks of the previously introduced solutions for triggering.

	Audio	$\omega_{\mathrm{energy}}+j$	$\omega_{y}+j$
**Advantage**	-	high sensitivity, most of the shots are detected as the swing in all axes is captured	high selectivity, false shots are very rare, for example grabbing a ball from the ground by hitting the racket on it
**Drawback**	impact not distinguishable from wind	lacks in selectivity, too many false positives	some shots are not detected due to slow angular velocities in the *y*-direction
